# Idiosyncratic Drug-Induced Liver Injury and Trastuzumab Deruxtecan in Breast Cancer: A Case Report

**DOI:** 10.3390/curroncol32110606

**Published:** 2025-10-31

**Authors:** Camilla Lisanti, Serena Della Rossa, Emma Zottarelli, Riccardo Vida, Silvia Bolzonello, Lucia Da Ros, Adrian Zdjelar, Erika Cecchin, Tiziana Perin, Fabio Puglisi

**Affiliations:** 1 Experimental and Clinical Pharmacology, Centro di Riferimento Oncologico di Aviano (CRO) IRCCS, 33081 Aviano, Italy; 2 Department of Medicine (DMED), University of Udine, 33100 Udine, Italy; serena.dellarossa@cro.it (S.D.R.); emma.zottarelli@cro.it (E.Z.); riccardo.vida@cro.it (R.V.); fabio.puglisi@cro.it (F.P.); 3 Department of Medical Oncology, Centro di Riferimento Oncologico di Aviano (CRO) IRCCS, 33081 Aviano, Italy; silvia.bolzonello@cro.it (S.B.); lucia.daros@cro.it (L.D.R.); 4 Department of Oncologic Radiation Therapy and Diagnostic Imaging, Centro di Riferimento Oncologico di Aviano (CRO) IRCCS, 33081 Aviano, Italy; adrian.zdjelar@cro.it; 5 Pathology Unit, Centro di Riferimento Oncologico di Aviano (CRO) IRCCS, 33081 Aviano, Italy; tperin@cro.it

**Keywords:** trastuzumab deruxtecan, HER2-positive breast cancer, drug-induced liver injury, hepatotoxicity

## Abstract

**Simple Summary:**

Trastuzumab deruxtecan (T-DXd) is an antibody–drug conjugate approved for the treatment of HER2-positive and HER2-low metastatic breast cancer. While its efficacy has been well demonstrated across several clinical trials, its safety profile—particularly with regard to hepatotoxicity—remains an area of emerging concern. In this case report, we describe a rare and severe episode of drug-induced liver injury (DILI) in a woman treated with T-DXd. After only two treatment cycles, the patient developed persistent grade 3 liver enzyme elevations, with biopsy findings confirming hepatocellular damage and prompting treatment discontinuation. This case underscores the importance of regular liver function monitoring during T-DXd therapy and highlights the need for increased clinical awareness of potential hepatotoxic effects, even in asymptomatic patients. As the use of T-DXd continues to expand, clinicians should remain vigilant for rare but serious adverse events such as DILI to ensure timely diagnosis and management.

**Abstract:**

Trastuzumab deruxtecan (T-DXd) has demonstrated efficacy in HER2-positive and HER2-low breast cancer. Its main safety concern is interstitial lung disease, while clinically relevant hepatotoxicity is rarely reported. In our case report we describe a 49-year-old woman with HER2-positive advanced breast cancer that developed persistent grade 3 transaminase elevations after 2 cycles of T-DXd, refractory to corticosteroid treatment and requiring treatment discontinuation. This case underlines the unpredictable and idiosyncratic nature of T-DXd associated hepatotoxicity and the importance of liver function monitoring. Clinicians should consider DILI in patients with unexplained liver enzyme elevations during therapy. Further studies are needed to clarify mechanisms and risk factors.

## 1. Introduction

Trastuzumab deruxtecan (T-DXd) is an antibody–drug conjugate (ADC) composed of a humanized anti-HER2 monoclonal antibody conjugated to a topoisomerase I inhibitor via a cleavable tetrapeptide linker. Its unique structure allows for the targeted delivery of high concentrations of cytotoxic payload to HER2-expressing cells and adjacent cells through a bystander effect [1]. T-DXd’s efficacy was initially evaluated in tumors with high HER2 membrane expression. In the phase II DESTINY-Breast01 (DB-01) trial [2], T-DXd demonstrated impressive results in heavily pretreated patients with HER2-positive metastatic breast cancer (mBC), showing a median progression-free survival (mPFS) of 19.4 months and a median duration of response (mDoR) of 20 months. The subsequent phase III DESTINY-Breast03 (DB-03) trial [3] further confirmed the superiority of T-DXd over T-DM1 in second-line therapy for HER2-positive mBC patients, significantly improving both mPFS and overall survival (OS) after progression on first-line therapy with a taxane, trastuzumab, and pertuzumab therapy. Moreover, the DESTINY-Breast02 (DB-02) trial demonstrated that T-DXd significantly improved both PFS and OS compared to physician’s choice in patients with HER2-positive unresectable and/or metastatic breast cancer previously treated with T-DM1 [4]. In addition, preliminary data from the DESTINY-Breast09 (DB-09) trial suggest that T-DXd may have substantial activity as a first-line option for HER2-positive mBC, further broadening its clinical utility [5].

Importantly, the therapeutic potential of T-DXd has expanded beyond HER2-positive disease. The phase III DESTINY-Breast04 (DB-04) trial represented a paradigm shift by demonstrating a significant improvement in both PFS and OS in patients with HER2-low mBC, defined as tumors exhibiting HER2 immunohistochemistry (IHC) scores of 1+ or 2+ without HER2 gene amplification [6]. Eligible patients had previously received standard endocrine therapy and at least one line of chemotherapy. Building upon these findings, the DESTINY-Breast06 (DB-06) trial further demonstrated the efficacy of T-DXd in HER2-low mBC in earlier lines of treatment, significantly outperforming standard chemotherapy right after progression on endocrine-based regimens [7].

Based on these data, T-DXd is currently approved for the treatment of patients with unresectable or metastatic HER2-positive breast cancer who have received a prior anti-HER2-based regimen either in the metastatic setting or in the (neo)adjuvant setting and have experienced disease recurrence during or within six months of completing therapy. It is also approved for patients with unresectable or metastatic HER2-low breast cancer who have received prior chemotherapy in the metastatic setting or experienced recurrence during or within six months of completing adjuvant chemotherapy.

In parallel with the efficacy data, the safety profile of T-DXd has been thoroughly evaluated in these trials. The drug was generally quite tolerated; the most common adverse events reported are gastrointestinal ones, such as nausea, vomiting and diarrhea; asthenia and alopecia. Interstitial lung disease (ILD) emerged as a notable safety concern, emphasizing the importance of monitoring for pulmonary toxicity in clinical practice. While ILD is a known risk, reports of hepatotoxicity with T-DXd are less common but clinically significant.

Drug-induced liver injury (DILI) is typically classified as acute or chronic based on the duration and histological pattern of liver damage and it can present in hepatitic, cholestatic, or mixed forms. The hepatitic form, characterized by hepatocyte necrosis, is often associated with a poor prognosis. Acute cholestatic DILI can present in several ways, ranging from bland cholestasis, which involves impaired biliary secretion without significant hepatocellular damage, to cholestatic hepatitis, where there is concurrent liver parenchymal injury, or a form involving bile duct injury or cholangitis [8].

This case report presents the case of a young woman with a metastatic HER2-positive breast cancer, who experienced DILI under treatment with T-Dxd.

## 2. Case Presentation

We describe the case of a pre-menopausal 49-years old woman, without a familiar oncological history, who was referred from her family doctor to a mammography and ultrasonography of the breast in July 2021 for the sudden onset of enlargement and increased tenderness of her right breast in June 2021 with a consensual nipple retraction. At the radiological evaluation, the findings described were compatible with carcinomatous mastitis. The core breast needle biopsy and the cytological evaluation of the axillary lymph node revealed an infiltrative, non-special-type, grade 3, HER2-positive (immunohistochemical score 3+), hormone receptors (HR) negative breast carcinoma, associated with numerous atypical and frank neoplastic cells in the ipsilateral axillary lymph node. Staging (18)F-fluorodeoxyglucose positron emission tomography/computed tomography (18F-FDG PET/CT) confirmed the locally advanced stage of the disease, with no evidence of distant secondary localizations.

Considering the biological characteristics and the initial stage of the disease, as well as the good general condition, after the discussion of the case at the multidisciplinary board, a neoadjuvant treatment with weekly paclitaxel, trastuzumab and pertuzumab was proposed, which the patient received from August 2021 to February 2022, with good tolerance.

The breast magnetic resonance imaging (MRI) and the bilateral echo-mammographic control performed at the end of the neoadjuvant treatment had documented a radiological partial response; therefore, after a new discussion of the case at the multidisciplinary board, radical right mastectomy was proposed to the patient and performed on 14 March 2022. The histopathological evaluation of the surgical specimen revealed only rare foci of intraductal carcinoma and a micro metastasis in one of the twelve removed axillary right lymph nodes.

Adjuvant radiotherapy on the right chest wall and ipsilateral lymph node drainages at the dose of 40.05 Gy in 15 fractions was subsequently delivered with an overall good tolerance and no significant toxicities. According to presurgical stage, residual disease, her good performance status, participation in the phase III, randomized, placebo-controlled, double-blind clinical trial ASTEFANIA was proposed: this trial evaluated the efficacy and safety of adjuvant atezolizumab or placebo in combination with T-DM1 for HER2-positive breast cancer at high risk of recurrence following preoperative therapy. The patient accepted the proposal and subsequent randomization had assigned her to the treatment arm with T-DM1 in combination with atezolizumab/placebo.

However, clinical study treatment was discontinued early, after 2 cycles of therapy, due to the occurrence of grade 2 (G2) hyperbilirubinemia. The abdominal ultrasound did not reveal any alterations suspicious for disease recurrence, nor signs of cholestasis, but did report the appearance of alterations compatible with initial hepatic steatosis. Considering the reported toxicity, after normalization of bilirubin value, the patient was proposed to continue adjuvant treatment with trastuzumab, at the standard dosage and schedule, to complete the year of anti-HER2 therapy, concluded in February 2023. No further liver toxicity was detected during trastuzumab treatment.

One month after completing adjuvant treatment, the patient visited the emergency room due to the onset of cerebral obtundation, mental confusion, dysarthria and recurrent headache; the brain CT scan showed the presence of six bilateral brain lesions compatible with secondary lesions, surrounded by significant perilesional edema. After neurosurgical evaluation, with exclusion of any indications to a surgical approach, an anti-edema intravenously therapy was started with mannitol and dexamethasone. After transfer to our Institute, a brain MRI was performed and confirmed the presence and extent of the brain disease; a subsequent radiotherapeutic consultancy was performed, with indication for stereotactic radiotherapy on single brain lesions, performed in the following days. A restaging CT scan of thorax and abdomen was performed, without evidence of other secondary lesions.

After collegial discussion of the case, given the actual stage and the biological characteristics of disease, the recurrence timing, a first line therapy with T-DXd was proposed, of which the first cycle was administered on 4 March 2023. After the first cycle, administered at the standard dose of 5.4 mg/kg, a dose reduction to 4.4 mg/kg was required for the second cycle, due to grade 3 (G3) asthenia, G2 anorexia and G3 oral mucositis.

After the administration of the second cycle of T-DXd, a progressive and significant increase in liver enzymes was recorded, in particular aspartate transaminase (AST) and alanine transaminase (ALT), up to persistent grade 3 toxicity, in the absence of associated symptoms; grade 1 hypoalbuminemia was associated, while other indices of liver function and cholestasis, including coagulation tests, showed no alterations.

**Figure 1:**
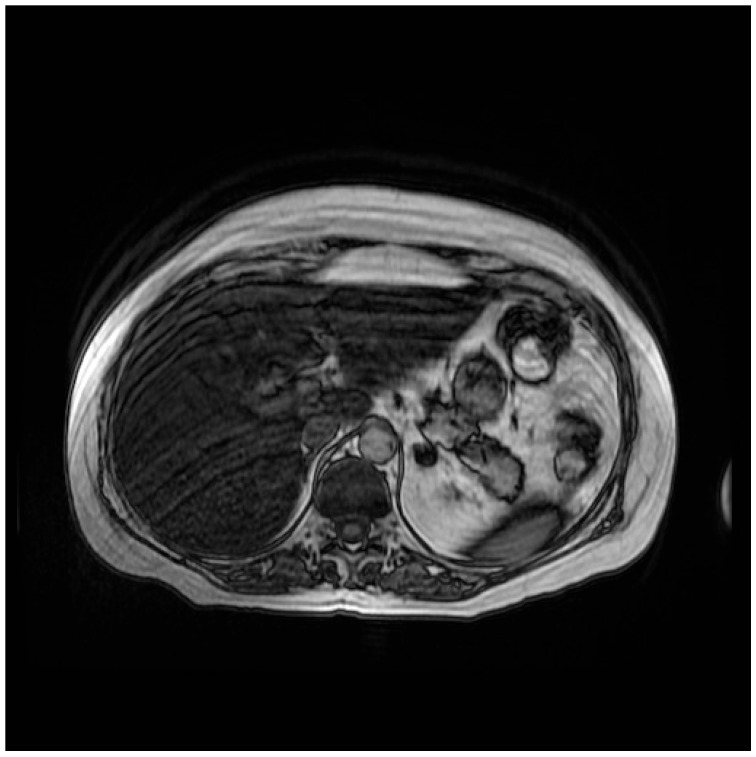
Abdominal MR sequence out-of-phase.

**Figure 2:**
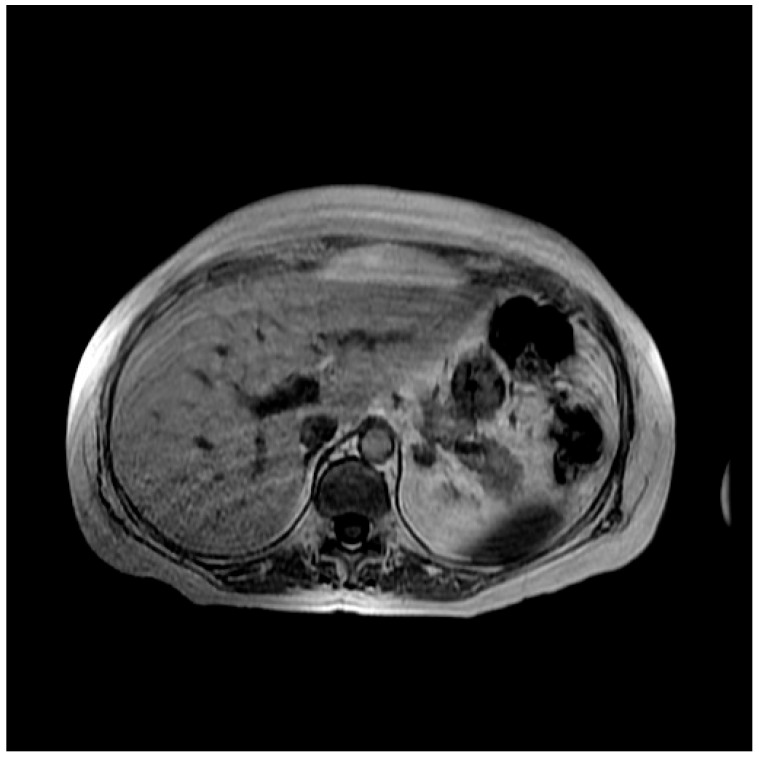
Abdominal MR sequence in-phase.

In order to determine the cause of these alterations, first a liver MRI was performed, which highlighted the appearance of severe steatosis (Figure 1 and Figure 2). In light of the MRI results, a hepatology consultation was requested, which recommended performing a complete laboratory evaluation that included both viral serology potentially involved in liver damage (such as hepatitis A virus, hepatitis B virus, hepatitis C virus, hepatitis D virus, Citomegalovirus, Epstein–Barr virus, Toxoplasma gondii), and indices potentially associated with autoimmune hepatitis conditions; all the above-mentioned tests were negative. On the advice of the hepatologist, steroid therapy, started for anti-edema purposes due to brain metastases, was continued.

**Figure 3:**
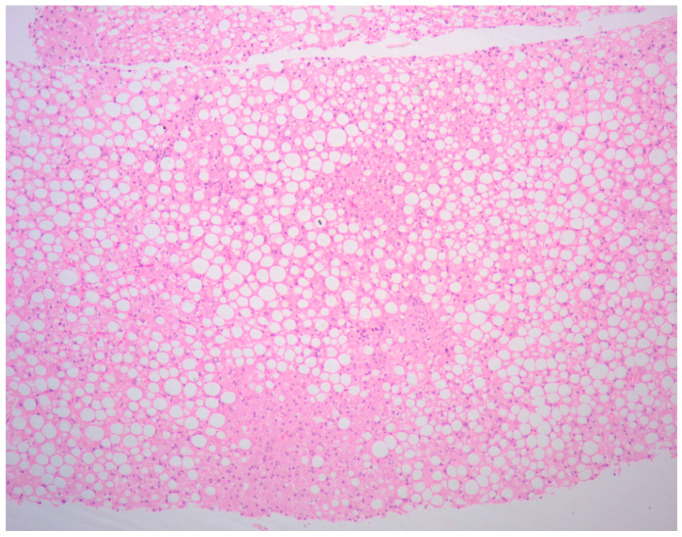
Microvesicular steatosis of hepatocytes (×5).

**Figure 4:**
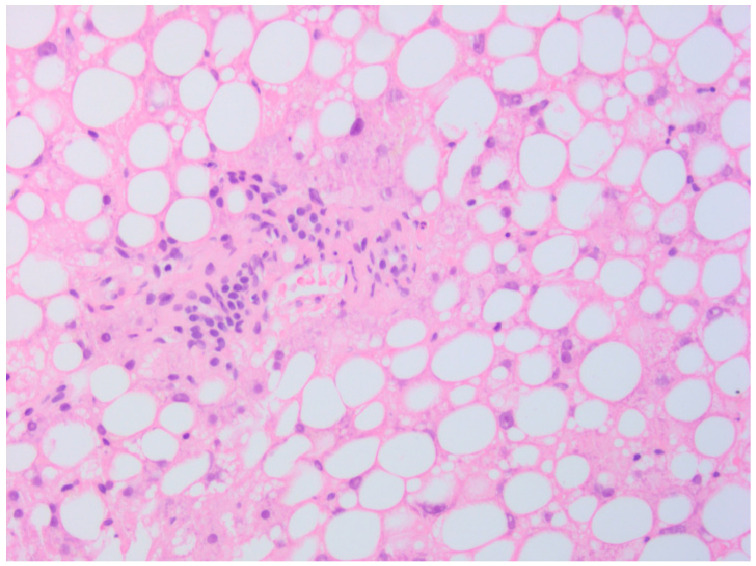
Microvesicular steatosis of hepatocytes (×25).

Since no possible cause responsible for the grade 3 alterations of liver enzymes (AST and ALT) and grade 1 hypoalbuminemia, persistent and initially poorly responsive to steroid therapy, had been identified up to that point, after a new consultation with the hepatologist, the execution of randomized ultrasound-guided liver needle biopsies was also agreed with the patient. The histological examination of the liver biopsies described the presence of “microvesicular steatosis but not exclusive, “cloudy” degeneration of hepatocytes”, findings compatible with DILI not specifiable if totally or partially reversible (Figure 3 and Figure 4).

**Figure 5:**
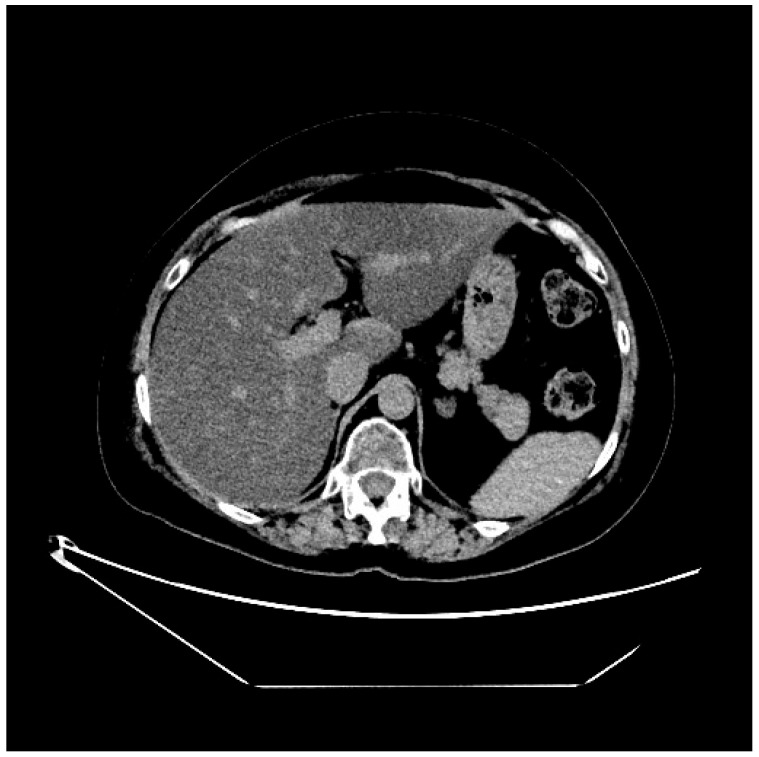
Abdominal CT scan.

**Figure 6:**
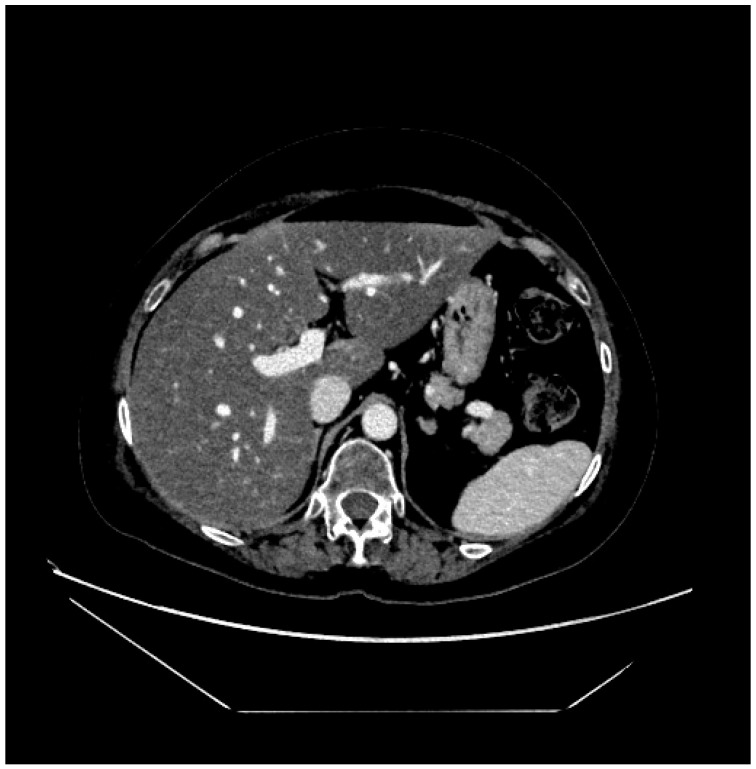
Abdominal CT scan.

Therefore, in light of what has emerged and the radiological confirmation of liver damage also in the abdominal CT scan repeated 1 month after the abdominal MRI (Figure 5 and Figure 6), it was collectively agreed to definitively discontinue treatment with T-DXd and to continue with the best supportive care to promote liver function tests recovery.

Approximately 3 months after the last administration of T-DXd, upon completion of steroid tapering and with the reduction in liver enzymes (AST and ALT) to grade 1 and hypoalbuminemia to grade 0, the patient was offered to start treatment with carboplatin and trastuzumab every 21 days. After the administration of the first cycle of carboplatin and trastuzumab, a new worsening of AST and ALT values was found (grade 2) with slow and progressive subsequent recovery to grade 1; so, we decided to continue treatment only with trastuzumab every 21 days, without further significant alterations in liver enzymes.

The head-thorax-abdomen CT scan, performed on 7 March 2024, had highlighted a progression of the disease in the thoracic lymph nodes, with suspected infiltration of the esophagus; therefore a second-line treatment was started with weekly paclitaxel (at a dose reduced to 75% given the persistent grade 1 alteration of AST and ALT), and trastuzumab every 21 days, which the patient received for only 2 months, suspending it at the end of October 2024 due to a marked worsening of the general clinical conditions and the detection of thoracic progression of the disease at the re-evaluation CT scan performed. The patient died in December 2024 at home, followed by the palliative care team.

## 3. Discussion

The case reported highlights an important and rare instance of severe liver dysfunction associated with T-DXd, classified as DILI. While DILI is a known adverse event for many cancer therapies, this is, to our knowledge, one of the first documented cases of severe DILI specifically linked to T-DXd. Given the increasing use of T-DXd across HER2-positive and HER2-low breast cancer as well as in other HER2 expressing malignancies, this case underscores the need for heightened clinical awareness and vigilance regarding potential hepatotoxic adverse events.

HER2-positive breast cancer accounts for up to 20% of diagnosed breast cancer cases, typically associated with an aggressive disease course, poor prognosis, and high rates of recurrence [9,10]. The therapeutic landscape for HER2-positive metastatic breast cancer (mBC) has undergone an impressive transformation with the advent of T-DXd, an ADC that has shown remarkable clinical efficacy in patients who have progressed on prior anti-HER2 therapies [11].

However, data from pivotal phase III trials reveal that T-DXd is associated with certain treatment-emergent adverse events (TEAEs), including hepatotoxicity. In the DB-03 trial, the incidence of grade ≥ 3 TEAEs was 56% in patients treated with T-DXd, comparable to the 52% in the T-DM1 group. Liver enzyme elevation was observed in registrational trials however was not necessarily higher when compared to control arms: for instance, in the DB03 trial elevated AST was reported in 28% of patients receiving T-DXd, compared to 41% in the T-DM1 group, and ALT elevations occurred in 23% of T-DXd-treated patients versus 32% in the T-DM1 cohort [3].

In Table 1 all Grade and Grade 3 or higher incidence rates of transaminase elevation are reported in some pivotal trials evaluating T-DXd in breast, colorectal, gastric and lung cancer. Data are reported for the overall population enrolled in the trials, irrespective of the presence/absence of liver metastases.

More detailed pharmacovigilance studies have raised concerns about T-DXd-associated hepatotoxicity. A report from the FDA Adverse Event Reporting System (FAERS) identified 504 cases of DILI linked to ADCs, including T-DM1 and T-DXd. Specifically, 33 reports of DILI associated with T-DXd were documented between 2020 and the third quarter of 2022, with most cases reported from Japan (60.6%) [12].

These findings emphasize the need for careful monitoring of liver function in patients receiving T-DXd, especially given the potential for idiosyncratic DILI, which is unpredictable and often unrelated to dose or pharmacological mechanisms.

DILI is defined as liver damage caused by medications or other xenobiotics, presenting as abnormal liver function tests or overt liver dysfunction, with other causes reasonably excluded.

Typically, it is characterized by an elevation in ALT levels exceeding five times the upper limit of normal (ULN), an increase in alkaline phosphatase (ALP) levels beyond two times the ULN, or a concurrent elevation of ALT to three times ULN accompanied by bilirubin levels more than twice the ULN. These abnormalities must occur within 90 days of starting the suspected drug and resolve within one month of discontinuation [13].

In clinical practice, two predominant biochemical patterns ca be delineated: hepatocellular and cholestatic, the former is suggested by rises in aminotransferases, reflecting leakage from damaged hepatocytes; the latter is characterized by elevated ALP and may coexist with hepatocellular injury. In both patterns, increased total bilirubin (conjugated + unconjugated) signals impaired hepatic excretory function and portend greater severity [14].

DILI can be classified as predictable (dose-dependent and related to the direct toxicity of the drug or its metabolites) or idiosyncratic (unpredictable, dose-independent) [15]. Most cases of DILI are idiosyncratic, believed to result from interactions between the drug and host-specific factors such as inflammation, immune responses, or genetic predispositions [16,17].

The hypothesis of an immune-mediated mechanism for idiosyncratic DILI has been reinforced by consistent links between specific HLA alleles and drug susceptibility, suggesting that hepatic biotransformation yields reactive metabolites that covalently modify cellular proteins, generating drug–protein adducts which at the end lead to the activation of the adaptive immune response [18]. T-DXd-related hepatotoxicity is likely idiosyncratic, given its unpredictable nature.

In our case, the patient exhibited poor tolerance to T-DXd, with liver enzyme elevations developing after just two treatment cycles. This timeline, along with the exclusion of infectious or autoimmune causes of liver injury, strongly suggests that T-DXd was the likely cause of DILI. Previous treatment, in the post-neoadjuvant setting, of the patient with T-DM1, a known cause of hepatotoxicity with a LiverTox score of B, may have predisposed her to liver injury, although the exact contribution of previous therapies remains unclear at this time [12].

One of the challenges in diagnosing and managing DILI is the absence of specific diagnostic tests making the diagnosis made by exclusion. Hy’s law has been formalized to define DILI after exclusion of alternative cause [19]; however, the more recent definition of DILI does not require such stringent biochemical criteria and is broader [13]. Clinical assessment tools such as the Roussel Uclaf Causality Assessment Method (RUCAM) can aid in the diagnosis [20], though they have limitations, especially in patients exposed to multiple potentially hepatotoxic agents [21]. In addition, all these tools have not been developed and validated in the context of oncological patients, who represent a complex and specific population for several reasons: exposure to prior therapies, use of multiple comedications, potential presence of liver metastases as well as baseline comorbidities prior to cancer diagnosis (such as liver steatosis and/or MASH) [22]. Some recommendations for DILI assessment during clinical trials in oncology patients have been recently published but there is still much room for further research in this field [23].

Also, the role of liver biopsy is still under debate, since it is not mandatory for the diagnosis of DILI; nevertheless, some experiences have shown that biopsies can help in refining the likelihood of the initial diagnosis of DILI [24].

Some histopathological patterns have been described and more the 12 have been reported even though some of them are definitively more frequent: among them acute hepatitis (lobular hepatitis), chronic hepatitis (portal hepatitis), zonal necrosis, acute cholestasis (bland cholestasis), cholestatic hepatitis, chronic cholestasis, steatosis/steatohepatitis, nodular regenerative hyperplasia and sinusoidal obstruction syndrome (veno-occlusive disease) [25]. However, also these specific patterns are not pathognomonic and only in some cases they have been more strongly associated with specific drugs.

In this complex scenario, ADCs add an additional level of complexity for several reasons. First, because of timing and onset of alteration in laboratory exams; this is an important point in the diagnosis of a potential DILI as well as for the potential rechallenge of the drug; contrary to other drugs and chemotherapy agents, ADCs are characterized by a longer half-life, making even more difficult the temporal association to define a DILI. Second, because of the structure of the ADC, consisting of a monoclonal antibody, a linker and a payload. The precise mechanisms underlying the liver enzyme elevations seen with T-DXd remain unclear, though it is believed that the topoisomerase inhibitor component, rather than the monoclonal antibody itself, is responsible. According to LiverTox, trastuzumab itself is classified as a possible but rare cause of clinically apparent liver injury (Class D), even though hepatotoxicity was more frequently reported in combination with chemotherapy agents such as paclitaxel [26].

The literature documents several cases of trastuzumab-induced hepatotoxicity, establishing a causal relationship based on temporal association, transaminase normalization after drug discontinuation, and recurrence of liver injury upon rechallenge with trastuzumab. However, most cases occurred in the context of combination therapy, complicating the attribution of liver injury to trastuzumab alone [27,28,29,30,31].

Notably, there is currently no established classification for T-DXd in the LiverTox database, although its structure, which includes a topoisomerase I inhibitor (deruxtecan), likely contributes to its hepatotoxic potential.

Management of such toxicity has not been clearly established; in most cases, these enzyme elevations are transient and self-limited, not accompanied by clinical symptoms such as jaundice and therefore not requiring specific intervention. As for the role of corticosteroids in this context, it remains a topic of debate. On one hand, corticosteroids can be utilized to manage DILI, although their efficacy has not been definitively proven. On the other hand, high-dose corticosteroids, such as the dexamethasone regimen the patient received for anti-edema purposes (up to 8 mg twice daily), may lead to drug–drug interactions through cytochrome P450 induction, potentially resulting in elevated liver enzymes [32].

Given the limited data on T-DXd-related hepatotoxicity and the challenges in establishing causality, further studies are needed to clarify the incidence and mechanisms of liver injury in patients receiving this therapy. As the clinical use of T-DXd continues to expand, particularly in tumor types beyond breast cancer, increased pharmacovigilance and real-world evidence will be essential in identifying and managing this rare but potentially serious adverse event. Early detection and intervention, including dose modifications or drug discontinuation, may prevent progression to severe liver injury.

## 4. Conclusions

In conclusion, while T-DXd offers substantial therapeutic benefits for patients with HER2-positive cancers, this case illustrates the need for careful monitoring of liver function during treatment. Clinicians should remain vigilant for signs of hepatotoxicity and DILI should be considered in any patient presenting with abnormal liver enzymes during T-DXd therapy. Further research is warranted to better understand the risk factors and mechanisms of DILI in patients receiving T-DXd, ultimately improving their safety and clinical outcomes.

## Figures and Tables

**Table 1:** Incidence of transaminase elevation (all Grades and Grade ≥3) in pivotal trials of T-DXd in breast, colorectal, gastric, and lung cancer.

Disease	Trial °	Control Arm	Enzymes	aG Exp Arm	≥G3 exp arm	aG ctrl Arm	≥G3 Ctrl Arm
Breast	DB-03	T-DM1	AST	23.3	0.8	37.2	5.0
			ALT	19.5	1.6	27.2	4.6
	DB-04	TPC	TA	34.0	6.7	31.4	13.4
	DB-06	TPC	TA	29.5	2.3	11.8	0
	DB-09	THP	TA	36.0	4.5	18.8	2.1
Gastric	DG-01	TPC	TA	NR	NR	NR	NR
	DG-02	-	AST	15.2	1.3		
			ALT	8.9	1.3		
	DG-04	RAM + PTX	TA	21.7	2.0	9.4	0.4
Lung	DL-01 *	-	AST	7.3	0		
			ALT	98	0		
	DL-02 **	-	TA	21.8	3.8		
Colon	CRC-01	-	AST	7.7	2.6		
			ALT	7.7	1.3		
	CRC-02	-	AST	8.4	0		

aG: all grade adverse events; ≥G3: grade 3 or higher adverse events; exp: experimental; ctrl: control; DB: DESTINY-Breast; DG: DESTINY-Gastric; DL: DESTINY-Lung; CRC: DESTINY-CRC; TPC: treatment of physician’s choice; RAM: ramucirumab; PTX: paclitaxel; NR: not reported; TA: transaminases. ° For all the trials, except for gastric cancer, T-DXd was used at the dose of 5.4 mg/kg. * Data are reported for the HER2-amplified cohort receiving T-DXd at 5.4 mg/kg. ** Data are reported for the HER2-mutated cohort receiving T-DXd at 5.4 mg/kg.

## Data Availability

The dataset supporting the conclusion of this article is included within the article.

## Abbreviations

The following abbreviations are used in this manuscript:

T-DXd	Trastuzumab deruxtecan
DILI	Drug-induced liver injury
ADC	Antibody–drug conjugate
DB-01	DESTINY-Breast01
mBC	Metastatic breast cancer
mPFS	Median progression-free survival
mDoR	Median duration of response
DB-03	DESTINY-Breast03
T-DM1	Trastuzumab emtansine
OS	Overall survival
DB-02	DESTINY-Breast02
DB-09	DESTINY-Breast09
DB-04	DESTINY-Breast04
IHC	Immunohistochemistry
DB-06	DESTINY-Breast06
ILD	Interstitial lung disease
HR	Hormone receptors
18F-FDG PET/CT	18F-fluorodeoxyglucose positron emission tomography/computed tomography
MRI	Magnetic resonance imaging
G	Grade
CT	Computed tomography
AST	Aspartate transaminase
ALT	Alanine transaminase
TEAEs	Treatment-emergent adverse events
FAERS	FDA Adverse Event Reporting System
ULN	Upper limit of normal
ALP	Alkaline phosphatase
RUCAM	Roussel Uclaf Causality Assessment Method
